# Supplementary material for: Novel perspective on a conventional technique: Impact of ultra-low temperature on bacterial viability and protein extraction
Source: PLoS One. 2021 May 17;16(5):e0251640. doi: 10.1371/journal.pone.0251640 (PMC8128238; doi:10.1371/journal.pone.0251640)
Supplement: S1 Fig — Comparative analysis of -80°C and -20°C storage of E. coli biomass for (A) short-term, 120 mins; and (B) long-term, 24 h and 48 h revealed that the lysis efficiency is relatively higher with -80°C storage than -20°C. (DOCX) [file pone.0251640.s001.docx]

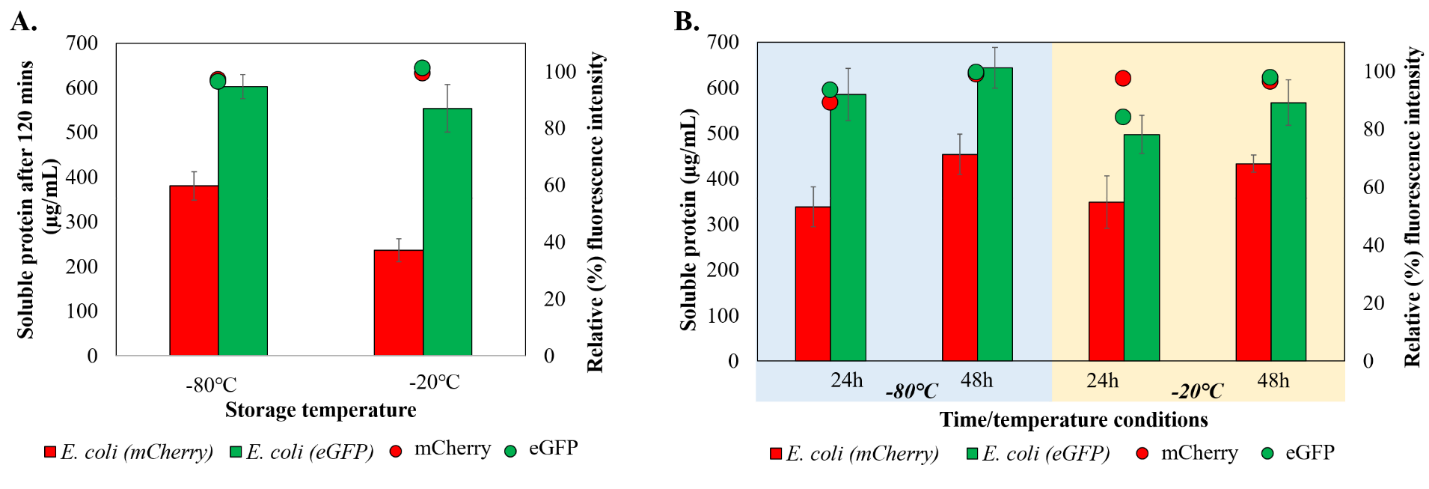


***S1 Fig.*** *Comparative analysis of -80℃ and -20℃ storage of E. coli biomass for (A) short-term, 120 mins; and (B) long-term, 24 h and 48 h revealed that the lysis efficiency is relatively higher with -80℃ storage than -20℃. Interestingly, both the storage temperatures yield higher protein concentration than the freshly harvested E. coli biomass with intact protein functionality indicated as relative (%) fluorescence intensities. Therefore, any of the temperatures can be used while practically implementing the strategy for improving the bacterial cell lysis, based on laboratory norms and accessibility.*
