# Supplementary material for: Novel perspective on a conventional technique: Impact of ultra-low temperature on bacterial viability and protein extraction
Source: PLoS One. 2021 May 17;16(5):e0251640. doi: 10.1371/journal.pone.0251640 (PMC8128238; doi:10.1371/journal.pone.0251640)
Supplement: S2 Fig — Effect of -20°C storage of E. coli biomass for short-term (120 mins) and long-term (24 h and 48 h) indicated that E. coli (mCherry) strains exhibited 4.6-folds improvement in the concentration of extracted protein after 48h, whereas E. coli (eGFP) showed 1.8-folds increase in the extracted protein concentration over that from the freshly harvested biomass. (DOCX) [file pone.0251640.s002.docx]

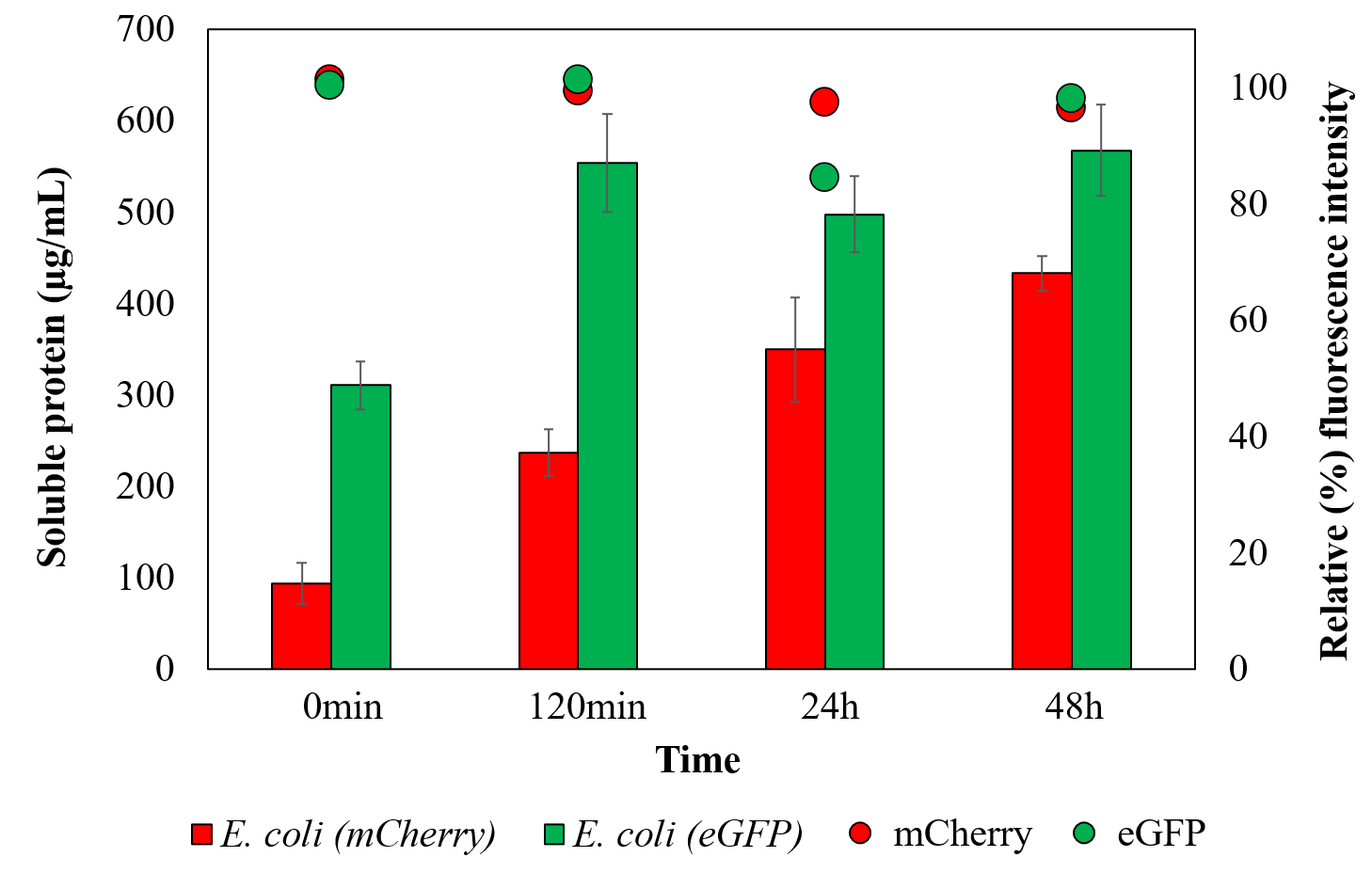


***S2 Fig.*** *Effect of -20℃ storage of E. coli biomass for short-term (120 mins) and long-term (24 h and 48 h) indicated that E. coli (mCherry) strains exhibited 4.6-folds improvement in the concentration of extracted protein after 48h, whereas E. coli (eGFP) showed 1.8-folds increase in the extracted protein concentration over that from the freshly harvested biomass. Relative (%) fluorescence intensity further supported our low temperature storage approach with sustained protein functionality. This experiment confirmed the practical implementation of -20℃ for improving the bacterial cell lysis.*
